# Age Differences in Associations Between Daily Emotional Support Provision and Memory Lapses

**DOI:** 10.1093/geroni/igaf122.3529

**Published:** 2025-12-31

**Authors:** Faith Shannon, Eric Cerino

**Affiliations:** Northern Arizona University, Flagstaff, Arizona, United States; Northern Arizona University, Flagstaff, Arizona, United States

## Abstract

Subjective cognitive complaints (e.g., memory lapses) may be an early warning sign of dementia detectable prior to declines in performance-based cognitive function. Identifying modifiable behaviors related to memory lapses is needed to target ways to promote cognitive health in everyday life. The role of emotional support provision, an interpersonal behavior requiring cognitive resources, has not been examined. We address this gap by assessing age differences in associations between emotional support provision and memory lapses. In 1,236 adults from the National Study of Daily Experiences (average age=67.67 years, SD = 10.34, 57% female), participants completed surveys for 8 days with questions regarding whether they gave emotional support and experienced retrospective or prospective memory lapses. We aggregated responses across days to calculate individual differences in proportion of days participants gave emotional support and reported memory lapses. Regression analyses adjusted for age, sex, education, race, caregiving status, and receipt of emotional support. People who gave more emotional support reported more retrospective (Est.=0.08, SE = 0.04, p = 0.02, 95% CI: 0.01, 0.16) and prospective memory lapses (Est.=0.14, SE = 0.03, p<.001, 95% CI: 0.08, 0.20). Age moderated the association between emotional support provision and prospective lapses (Est.=0.01, SE = 0.01, p<.01, 95% CI: 0.002, 0.01) such that the association between emotional support provision and prospective memory lapses was amplified in comparatively older adults (Est.=0.21, SE = 0.04, p<.001, 95% CI: 0.13, 0.30). Results indicate emotional support provision is linked to elevated risk of memory lapses, especially for comparatively older adults that may have less resources to manage demands of emotional support provision and prospective memory.

